# Metabolomics and biochemical analysis reveal the regulatory mechanism of exogenous sorbitol-chelated potassium on wheat under drought stress

**DOI:** 10.3389/fpls.2026.1751075

**Published:** 2026-03-18

**Authors:** Mingxia Zhang, Guohui Du, Huanyang Zhang, Ruili Zheng, Li Zhao, Mingli Huang, Xiaocui Wang, Kezhong Liu, Dongyun Yan

**Affiliations:** 1 College of Environmental and Geographical Sciences, Qingdao University, Qingdao, China; 2 Department of Food Industry, Shandong Institute of Commerce and Technology, Jinan, China

**Keywords:** antioxidant system, drought stress, metabolomics, sorbitol-chelated potassium, wheat

## Abstract

Potassium fertilization is a strategy to alleviate the impact of drought stress on wheat production. However, the effects of chelated potassium remain to be verified. This study simulated drought stress using 10% PEG-6000 (-0.8MPa) and investigated the effects of spraying with distilled water (CK_2_), sorbitol (S), potassium chloride (K), sorbitol mixed with potassium (MK), and sorbitol-chelated potassium (SK) on the biomass, photosynthetic performance, antioxidant system, osmoregulation capacity, and metabolome of wheat (*Triticum aestivum* L.) seedlings. The results showed that SK treatment alleviated the inhibitory effect of drought on growth, with the aboveground biomass increasing by 15.66% and 20.00% compared to the K and MK treatments, respectively (*P* < 0.05). Compared with MK, SK treatment significantly increased total chlorophyll content by 18.74% and reduced malondialdehyde content by 16.02%, while also enhancing antioxidant enzyme activity and the accumulation of osmoregulatory substances. Metabolomic analysis revealed that 51 differential metabolites (11 upregulated and 40 downregulated) were identified in SK .vs. CK_2_, mainly including (-)-Jasmonoyl-L-isoleucine, N-Acetyl-D-glucosamine, and (+)-Abscisic acid. These metabolites were primarily enriched in pathways such as α-linolenic acid metabolism, histidine metabolism, plant hormone signal transduction, carotenoid biosynthesis, and flavonoid biosynthesis. This study reveals the potential role of specific metabolic pathways in the development of drought tolerance in wheat, providing a novel perspective for physiological research on crop drought resistance.

## Introduction

Drought stress disrupts key physiological processes in plants, impairing photosynthesis and antioxidant defenses (Qiu et al., 2025), and is a major factor limiting wheat growth and yield (Li J. P. et al., 2024). As an early response to water deficit, photosynthesis is notably vulnerable, with drought-induced damage to photosynthetic pigments, reductions in net photosynthetic rate, stomatal conductance, and transpiration rate, these ultimately disrupt plant metabolism and suppress biomass accumulation (Pirasteh-Anosheh et al., 2016; Li et al., 2017). In parallel, drought stress induces overproduction of reactive oxygen species (ROS), causing oxidative damage to plant cells and tissues. To mitigate the oxidative damage of ROS to cells and maintain redox balance, plants activate enzymatic antioxidant systems, which are composed of peroxidase (POD), superoxide dismutase (SOD), catalase (CAT), and glutathione peroxidase (GPX), and non-enzymatic antioxidant systems, including secondary metabolites such as flavonoids (FC) and total phenols (TPC) compounds (Foyer and Noctor, 2005; Hasanuzzaman et al., 2020). Additionally, ROS not only function as effector molecules in oxidative stress but also play a role in signal transduction. For example, ROS molecules and plant hormones such as auxin and abscisic acid may play a crucial role in perceiving stress signals, including drought and mechanical stress (Shahzad and Hameed, 2025).

Plant responses to drought by profiling metabolite changes. Serving as a bridge between plant genotype and phenotypes, the metabolome provides insights into mechanisms underlying phenotypic variation under stress conditions (Yang et al., 2019). Wang K. et al. (2024) using LC -MS technology, four key types of differential metabolites, namely amino acids, organic acids, sugars, and alkaloids, such as gibberellin A4 (GA4), abscisic acid (ABA), and sucrose, have been identified. These metabolites are crucial for alfalfa to withstand drought stress. Bowne et al. (2012) investigated a targeted GC-MS approach to monitor 103 structurally identified metabolites from leaf tissue of the drought-stressed wheat plants, predominantly amino and organic acids and sugars. However, most drought-stress studies have focused on individual plant systems, leaving the interactions among photosynthetic, antioxidant, osmotic adjustment and metabolomic responses underexplored.

Critical for maintaining growth and yield stability, potassium, a key macronutrient, enhances crop stress resistance by regulating stomatal movement, maintaining photosynthetic rates, detoxifying reactive oxygen species, and promoting the synthesis of soluble carbohydrates (e.g., proline and soluble sugars) (Ahanger and Agarwal, 2017; Hasanuzzaman et al., 2018; Johnson et al., 2022; Rawat et al., 2022). Drought stress restricts root growth and limits the mass flow and diffusion of available potassium in the soil toward the roots (Vetterlein and Jahn, 2004; Wang et al., 2013), making exogenous potassium application an effective strategy to compensate for the potassium deficiency and enhance drought tolerance. Sorbitol-chelated potassium, a novel chelated foliar potassium fertilizer, has demonstrated a positive regulatory role in improving wheat drought resilience. Sorbitol is a primary product of photosynthesis and possesses properties such as osmotic regulation, wetting, and surface tension reduction (Will et al., 2011), which make it particularly advantageous as a chelating ligand. Compared to conventional inorganic (non-chelated) potassium fertilizers, sorbitol-chelated potassium enhances potassium uptake, improves crop quality, and crop yield. In wheat, sorbitol-chelated potassium application has been reported to increase post-anthesis potassium accumulation and yield by 13.99%-35.48% (Han et al., 2025). Similar effects have also been reported in peanuts (Sun et al., 2024) and celery (Shi et al., 2024; Zhang et al., 2025). Therefore, the potential of sorbitol-chelated potassium to enhance stress resistance still needs further verification.

Despite these findings, studies investigating the effects of chelated potassium on crops under drought stress remain limited (Li et al., 2021). Given the role of potassium in wheat growth and stress responses, elucidating how sorbitol-chelated potassium enhances drought resistance holds significant scientific and practical implications. Because drought affects multiple physiological pathways, including photosynthesis, antioxidant defense, and metabolism, a systematic analysis integrating these factors is necessary to uncover potential mechanisms by which sorbitol-chelated potassium alleviates drought-induced damage in wheat.

## 2 Materials and methods

### 2.1 Plant material and environment

Plump XinHua818 wheat seeds of uniform size were selected and surface sterilized with 0.1% sodium hypochlorite for 10 minutes, followed by three rinses with ultrapure water. The sterilized seeds were incubated in the dark at 25°C for 24 hours in a biological incubator to promote germination.

Germinated seeds were placed into seedling trays lined with three layers of gauze and grown in a greenhouse under controlled conditions: temperature 25°C, light intensity 100 µmol m^-2^s^-1^, and a photoperiod of 16 h light/8 h dark. Once the seedlings reached 5–6 cm in height, they were transferred to plastic hydroponic containers (70 seedlings per container) containing Hoagland nutrient solution. The solution volume in each container was maintained consistently and replaced every 3 days.

### 2.2 Experimental design and drought treatment

**Figure 1:**
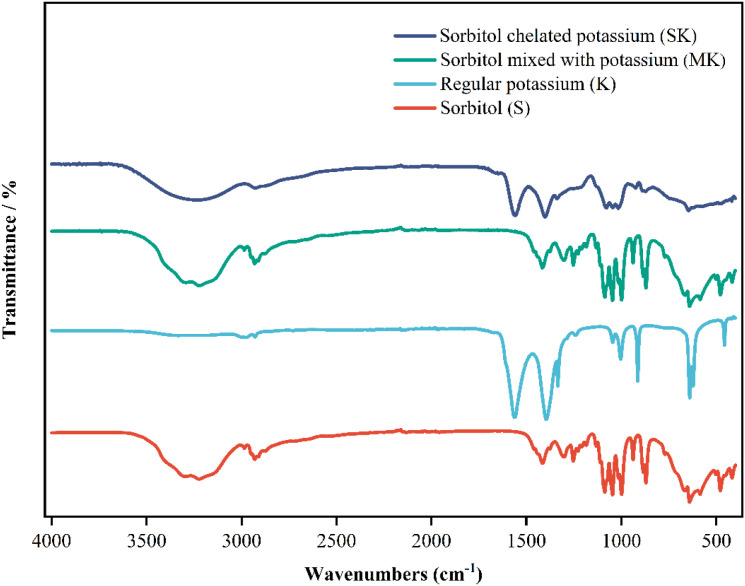
Fourier-transform infrared spectra (FT-IR) of sorbitol-chelated potassium.

At the two-leaf and one-heart stage, seedlings were transferred to hydroponic containers (8.5 cm diameter, 12 cm height) to commence experimental treatments. Six treatments were established; each treatment comprised three independent biological replicates. (48 seedlings per replicate). The normal control group (CK_1_) was supplied with Hoagland nutrient solution, while drought stress was simulated by supplementing the nutrient solution with 10% polyethylene glycol (PEG-6000, -0.80MPa), and a corresponding drought stress control group (CK_2_) was included. The experimental design is detailed in **Table 1**. The foliar fertilizer used in the experiment was self-prepared sorbitol-chelated potassium. The chelation ratio (molar ratio) was sorbitol to potassium chloride = 1:2. The water bath temperature was maintained at 75 °C, and the chelation time was 60 minutes. The chelation rate was 98.57%, and the pH was 5.87. Synthesized from potassium chloride (analytical purity ≥99.5%, Jiangsu Qiangsheng Functional Chemistry Co., Ltd.) and sorbitol (content ≥70%, China National Medicines Co., Ltd.). Foliar application of the corresponding reagents was performed on the seedlings under each treatment on Day 1 and Day 3 during the 7-day drought stress period. After the stress treatment, seedling samples were collected and immediately frozen in liquid nitrogen for subsequent analysis. After preparation and separation (Cui et al., 2021; Yan, 2021), the Fourier-transform infrared spectrum of the fertilizer was obtained (**Figure 1**).

**Table 1:** Experimental setup with different foliar spray treatments.

Treatment	Spraying concentration (mg·L^-1^)	Growth environment
Control (CK_1_)	–	Hoagland nutrient solution
Control (CK_2_)	Distilled water	Hoagland nutrient solution+10%PEG-6000
Sorbitol (S)	10 (K^+^)	
Potassium Chloride (K)		
Potassium Mixed with Sorbitol (MK)		
Sorbitol-chelated Potassium (SK)		

The spray concentration for potassium-containing treatments is based on the potassium ion (K^+^) concentration, while the sorbitol treatment is applied at the same sorbitol ligand concentration as present in both the sorbitol-mixed potassium and sorbitol-chelated potassium solutions.

The infrared spectroscopy results indicate that pure potassium ionic compounds should exhibit almost no absorption peaks in the infrared region. The physical mixture of sorbitol and potassium nitrate primarily shows a simple superposition of characteristic peaks. In contrast, for sorbitol-chelated potassium, in the O–H stretching vibration region (3600–3000 cm^−1^), peak shifts and broadening are observed. Furthermore, in the fingerprint region (1500–800& cm^−1^), which includes C–O stretching vibrations and O–H bending vibrations, distinct peak displacements occur. These changes demonstrate that the interaction between sorbitol and potassium ions has been altered, resulting in the formation of a new, stable structural environment.

### 2.3 Indicators and methods

#### 2.3.1 Growth indicators and leaf relative water content

For each treatment, twelve wheat plants were selected, washed with pure water, and wiped dry before measuring plant height and stem diameter. Each whole plant was then divided into above-ground and below-ground parts, and immediately weighed, with the weights recorded as root fresh weight and leaf fresh weight (FW). The leaves were soaked in double-distilled water for 24 h and then removed. After removing residual water from the leaf surface with absorbent paper, the leaves were weighed and the weight recorded as turgid weight (TW). The leaves were then deactivated at 105 °C for 30 min and dried at 60 °C to a constant weight, which was recorded as dry weight (DW). The relative water content of the leaves was calculated via the following formula: *RWC (%) =(FW-DW)/(TW-DW) ×100%* (Wu et al., 2020). Three biological replicates were analyzed.

#### 2.3.2 Determination of potassium content in plants

Determination of potassium content in plants and roots: Approximately 0.2 g of sample was weighed and subjected to microwave digestion using HNO3-H2O2 (6:1) (Touchwin2.0-GS25, Aople, Chengdu). The potassium content was then measured by ICP-OES (Avio 200, PerkinElmer, Massachusetts, USA).

#### 2.3.3 Chlorophyll content and gas exchange

Following the methods of Lichtenthaler and Wellburn (1983), with slight modifications, the first fully expanded leaf of wheat seedlings was selected, ground with a mortar, and extracted with acetone. The absorbance of chlorophyll *a*, chlorophyll *b* and carotenoids were measured at OD_663_, OD_645_ and OD_480_ nm using a spectrophotometer.

Following drought stress, the net photosynthetic rate (Pn), stomatal conductance (Gs), transpiration rate (Tr), and intercellular CO_2_ concentration (Ci) were determined using an Li-6800 porTable photosynthesis system (Li-CORBiosciences, Lincoln, NE, USA), as previously described by He et al. (2025). The measurements were conducted on the last fully expanded leaf, employing a photon flux density of 1000 μmol photo m^−2^ s^−1^ and a CO_2_ concentration of 400 μmol mol^−1^, within the time from of 9:00 a.m. to 11:00 a.m.

#### 2.3.4 Reactive oxygen species and antioxidant enzyme activity

The content of superoxide anion (O_2_^-^), superoxide dismutase (SOD), peroxidase (POD), and catalase (CAT) were determined using assay kits (Boxbio, Beijing) according to the manufacturer’s instructions. One gram of leaf sample was homogenized in 1 mL of extraction buffer in an ice bath, followed by centrifugation at 8,000 g for 10 min at 4 °C. The supernatant was then collected and used for reactive oxygen species and enzyme activity assays.

#### 2.3.5 Determination of malondialdehyde content and osmotic adjustment substance

Following the method outlined by Tan et al. (2008), a 1 g sample was homogenized with 10 mL of a mixed Trichloroacetic Acid-Thiobarbituric Acid (TCA-TBA) solution, followed by centrifugation at 4000 rpm for 10 min. The mixture was kept in a boiling water for 15 minutes and centrifuged at 4,000 rpm for 10 min. Malondialdehyde (MDA) content in the reaction solution was determined using a spectrophotometer at OD_450_, OD_532_ and OD_600_ nm.

Proline content was determined by the acid ninhydrin colorimetric method, and the absorbance of the extract was measured at a wavelength of OD_520_ (Bates et al., 1973). The soluble protein (SP) content was determined using Bradford (1976) Coomassie Brilliant Blue method. The supernatant was mixed with Coomassie Brilliant Blue G-250 for colorimetric measurement, and the absorbance was recorded at OD_595_. The soluble sugar (SS) content was determined by the anthrone–sulfuric acid method. Two portions of the supernatant were collected and mixed with 80% ethanol and anthrone reagent, heated in a boiling water bath for 10 minutes, cooled to room temperature, and the absorbance was measured at OD_630_ (Wang et al., 2022).

#### 2.3.6 Metabolite extraction

Six wheat seedlings were randomly selected for each treatment group. Leaves from the same position on each seedling were collected, immediately flash-frozen in liquid nitrogen, and then stored in a -40 °C freezer. Metabolomic analysis was performed only on the CK_2_, S, K, MK, and SK groups to evaluate the effects of exogenous treatments under drought stress. Each treatment included three biological replicates. Metabolomic analysis was conducted by Biomarker Technologies (Beijing, China) with the extraction procedure as follows:

(1) Weighed 50 mg of the sample into a pre-cooled mortar and grinded it into powder. The powder was then transferred to an EP tube, and 1000 μL of a mixed extraction solution of methanol, acetonitrile, and water (2:2:1, containing internal standard at 20 mg L^-1^) was added, followed by vortexing for 30 s.

(2) Steel beads were added, and the mixture was processed using a grinding instrument (45 Hz) for 10 min, followed by sonication in an ice-water bath for 10 min. Subsequently, the sample was allowed to stand at -20 °C for 1 h and then centrifuged at 12000 rpm (4 °C, 15 min).

(3) 500 μL of the supernatant was transferred to an EP tube and dried in a vacuum concentrator. The extract was then reconstituted with 160 μL of acetonitrile/water (1:1, v/v), vortexed for 30 s, and sonicated in an ice-water bath for 10 min.

(4) Collect 120 μL of the supernatant (12000 rpm, 4 °C, 15 min) into a 2 mL vial. A 10 μL aliquot from each sample was combined to prepare a quality control (QC) sample for instrument analysis. The QC sample was generated by mixing all test samples in equal proportions prior to mass spectrometry analysis. To evaluate potential errors caused by mass spectrometric signal drift, one QC injection was inserted after every ten samples during the analytical run to verify the stability of the entire detection process. Chromatographic conditions: An Acquity UPLC HSS T3 column (1.8 μm, 2.1*100 mm, Waters, US) was used with mobile phase A (0.1% formic acid in water) and mobile phase B (0.1% formic acid in acetonitrile). Chromatographic data acquisition was performed on an Acquity I-Class PLUS ultra-high-performance liquid chromatograph (Waters, US) coupled to a Xevo G2-XS QTOF (Waters, US) high-resolution mass spectrometer, with detection in both positive and negative ion modes.

### 2.4 Statistic analysis

Using SPSS 27.0 and Origin 2021 for one-way analysis of variance to determine differences between numerical values. Structural equation modeling (SEM) data processing was performed using SetupStata 18 and Power Point 2021 were used to generate. Using MassLynx V4.2 software to collect raw metabolomics data, the peaks were extracted and aligned through Progenesis QI software for data processing, and the Benjamini-Hochberg (BH) method (Benjamini and Yekutieli, 2001) was employed for p-value correction. Commercial databases, including the online METLIN database (based on Progenesis QI software), KEGG (Kyoto Encyclopedia of Genes and Genomes) (http://www.genome.jp/kegg/), HMDB (https://hmdb.ca), and Lipidmaps (http://www.lipidmaps.org/), were used to search metabolic pathways for profiling and metabolite identification. PCA and heatmap images were generated using MetaboAnalyst 6.0 (https://www.metaboanalyst.ca/) and Origin 2021.

## 3 Results

### 3.1 Effect of sorbitol-chelated potassium on wheat growth and biochemical indices under drought stress

#### 3.1 Biomass accumulation and potassium content

Different foliar spray treatments significantly affected the wheat seedling growth traits, including biomass, plant height, stem diameter, leaf relative water content (RWC),and plant potassium content (**Table 2**). Notably, sorbitol-chelated potassium treatment effectively promoted biomass accumulation compared to other drought stress treatments. Under drought stress, aboveground biomass in SK-treated seedlings increased by 15.66% and 20.00% compared to K and MK treatments, respectively (both significant). Root biomass also increased under SK treatment by 3.23% and 10.34% compared with K and MK treatments, respectively, though the difference was not significant relative to K treatment. Plant height increased by 8.42% under SK compared to K treatment, whereas stem diameter did not differ significantly across treatments. Additionally, SK treatment increased leaf RWC by 15.25% compared to K treatment, indicating enhanced water retention under drought stress. Under drought stress, the potassium content in the aboveground parts treated with SK increased significantly by 23.1% and 18.9% compared to the S and K treatments, respectively; compared with the S, K, and MK treatments, the potassium content in the roots treated with SK increased by 19.5%, 18.1%, and 8.3%, respectively.

**Table 2:** Growth indicators of wheat seedlings under normal conditions and drought stress with different fertilization treatments.

Treatment	Above-ground biomass (g)	Root biomass (g)	Plant height (cm)	Thick stem (mm)	Relative water content of leaves	Aboveground potassium content (mg·g^-1^)	Root potassium content (mg·g^-1^)
CK_1_	3.04 ± 0.09a	0.31 ± 0.00ab	23.46 ± 0.98a	2.46 ± 0.04a	0.88 ± 0.03a	62.79 ± 1.96b	24.81 ± 2.79a
CK_2_	2.42 ± 0.03b	0.28 ± 0.01c	22.23 ± 0.03ab	2.27 ± 0.04c	0.56 ± 0.03cd	55.82 ± 1.74c	26.33 ± 0.13a
S	2.47 ± 0.10b	0.28 ± 0.01c	22.29 ± 0.24ab	2.44 ± 0.03ab	0.47 ± 0.01d	55.52 ± 1.88c	23.26 ± 1.23a
K	2.49 ± 0.08b	0.31 ± 0.02ab	21.37 ± 0.26b	2.40 ± 0.00ab	0.59 ± 0.06bc	57.47 ± 1.79c	23.53 ± 0.50a
MK	2.40 ± 0.08b	0.290.00bc	22.42 ± 0.16ab	2.39 ± 0.02b	0.63 ± 0.06bc	65.73 ± 0.60ab	25.67 ± 2.22a
SK	2.88 ± 0.06a	0.32 ± 0.01a	23.17 ± 0.80a	2.40 ± 0.03ab	0.68 ± 0.05b	68.35 ± 0.75a	27.80 ± 0.34a

Different lowercase letters in the same column indicate significant differences between treatment groups for the same indicator (*p* < 0.05). (Above ground and root biomass measured as fresh weight)

#### 3.1.2 Photosynthetic pigments and gas exchange

**Figure 2:**
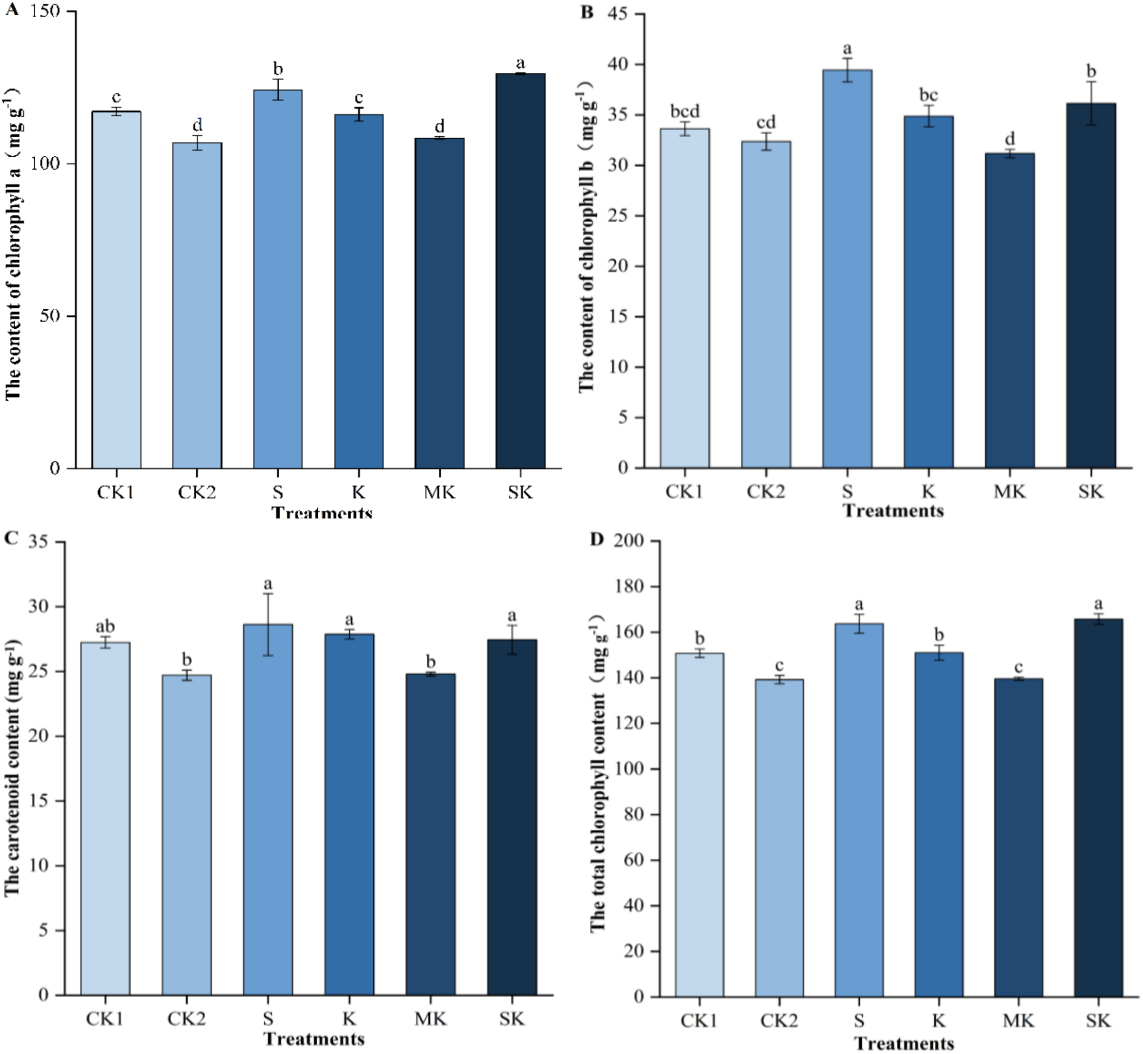
Changes in photosynthetic pigment content of wheat seedlings. Data are shown as mean ± SE. (n=3, biological independent replicate experiments). Different letters denote significant differences (*p* < 0.05). **(A)** changes in chlorophyll a content. **(B)** changes in chlorophyll b content. **(C)** changes in carotenoid content. **(D)** changes in total chlorophyll content.

Different foliar spray treatments significantly affected the accumulation of chlorophyll *a*, chlorophyll *b*, carotenoids, and total chlorophyll content in wheat seedlings under drought stress (**Figure 2**). Drought stress reduced photosynthetic pigment content, whereas foliar spraying alleviated this effect. Compared with the ionic potassium treatment, the sorbitol-chelated potassium treatment effectively increased chlorophyll *a* and total chlorophyll content, with significant differences. Chlorophyll *a* increased by 11.60% and 19.54% in SK treatment compared to K and MK treatments, respectively. Similarly, chlorophyll *b* and carotenoid content increased by 15.94% and 10.68%, respectively, compared with MK treatment. Carotenoid levels in SK treatment decreased by 1.52% and 4.11% relative to K and S treatments, respectively, though these differences were not statistically significant. Total chlorophyll content in SK treatment increased by 18.74% compared to MK treatment, a difference that was statistically significant.

**Figure 3:**
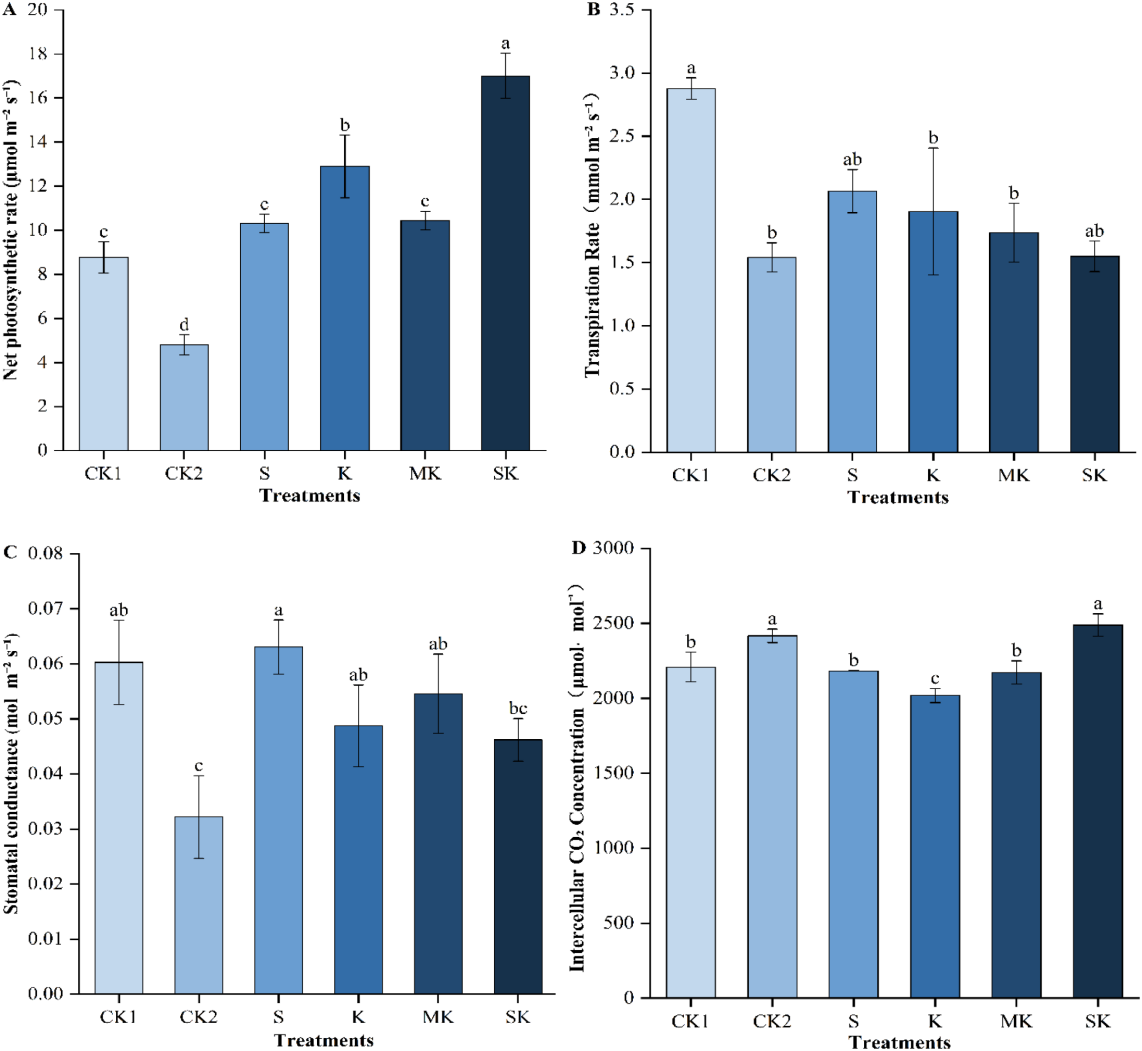
Changes in gas exchange of wheat seedling leaves. Data are shown as mean ± SE. (n=3, biological independent replicate experiments). Different letters denote significant differences (*p* < 0.05). **(A)** Net photosynthetic rate (Pn). **(B)** Transpiration rate (Tr). **(C)** Stomatal conductance (Gs). **(D)** Intercellular CO_2_ concentration (Ci).

Under drought stress, different spraying treatments significantly affected the net photosynthetic rate (Pn), transpiration rate (Tr), intercellular CO_2_ concentration (Ci), and stomatal conductance (Gs) of wheat seedlings (**Figure 3**). Drought stress decreased leaf gas exchange parameters relative to the control treatment (CK_1_). However, these parameters improved following foliar treatments. Compared with the MK, K, and S treatments, SK treatment significantly increased Pn by 62.90%, 31.98%, and 65.00%, respectively, and Ci by 14.55%, 23.28%, and 14.00%, respectively. Tr decreased by 18.64% and 25.00% under SK treatment compared to K and S treatments, respectively. However, these differences were not statistically significant. Gs decreased by 15.39% and 26.75% compared to MK and S treatments, respectively, with no significant

differences.

#### 3.1.3 Superoxide anion content and antioxidant system

**Figure 4:**
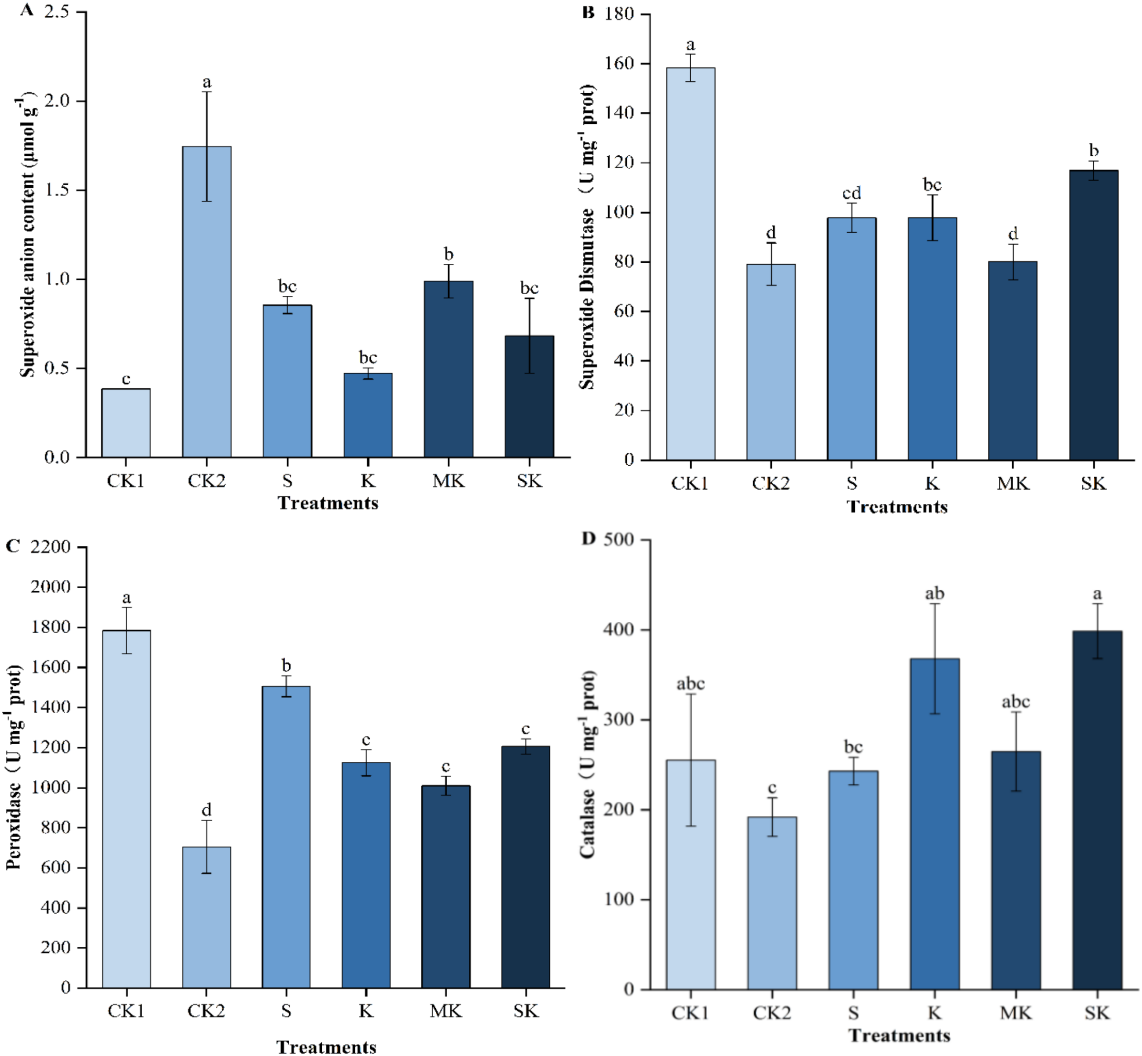
Superoxide anion content and antioxidant enzyme activity in wheat seedlings. Data are shown as mean ± SE. (n=3, biological independent replicate experiments). Different letters denote significant differences (*p* < 0.05). **(A)** Superoxide anion content (O_2_^-^), **(B)** Superoxide Dismutase (SOD), **(C)** Peroxidase (POD), **(D)** Catalase (CAT).

Under drought stress, the levels of superoxide anion content (O_2_^-^), superoxide dismutase (SOD), peroxidase (POD), and catalase (CAT) in wheat seedlings, were significantly affected by different foliar spray treatments (**Figure 4**). Drought stress leads to excessive accumulation of reactive oxygen species. After SK treatment, the O2- content was lower than that of MK and S treatments, with reductions of 30.91% and 20.10%, respectively. Drought stress significantly reduced SOD and POD activities. However, foliar application with sorbitol-chelated potassium (SK), potassium mixed with sorbitol (MK), sorbitol (S), potassium chloride (K) significantly increased SOD and POD activity compared to control treatment (CK_2_). SOD activity increased by 46.07% and 19.58% in SK treatment relative to MK and S treatments, respectively. CAT activity increased by 64.00% in SK treatment compared to S treatment. POD activity followed a similar trend, increasing by 7.23% and 19.46% in SK compared to K and MK treatments, respectively, but decreasing by 19.92% compared to S treatment.

#### 3.1.4 Osmoregulation system

**Figure 5:**
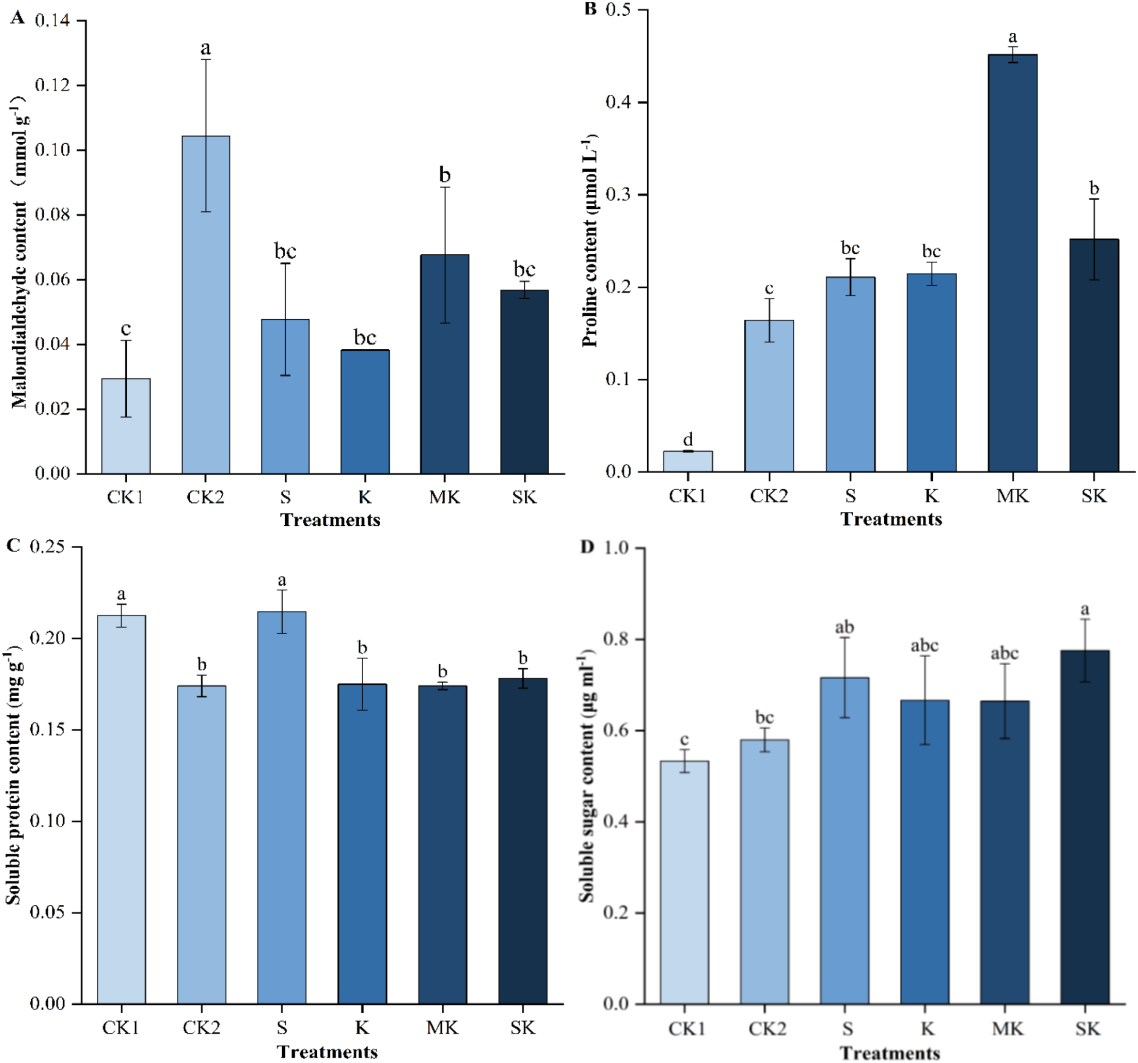
Changes in osmotic regulatory substances and malondialdehyde content in wheat seedling leaf cells. Data are shown as mean ± SE. (n=3, biological independent replicate experiments). Different letters denote significant differences (*p* < 0.05). **(A)** Malondialdehyde content (MDA), **(B)** Proline content (Pro), **(C)** Soluble protein (SP), **(D)** Soluble sugar content (SS).

Drought stress induced the accumulation of malondialdehyde (MDA) levels, enhancing membrane lipid peroxidation and causing damage to wheat seedlings (**Figure 5A**). The MDA levels was decreased by 16.02% in SK treatment compared to MK treatment. Compared with the MK treatment, the MDA content under the SK treatment decreased by 16.02%. To alleviate membrane damage, osmotic regulatory substances in the leaves accumulated significantly to maintain cellular homeostasis. Under drought stress, sorbitol-chelated potassium (SK) promoted the synthesis of proline (Pro) in leaves, increasing by 17.36% compared with inorganic potassium (K). Compared with CK_2_, all foliar fertilizer treatments under drought conditions increased the soluble protein (SP) content, with the SP content under SK treatment being 1.81% and 2.34% higher than those under K and MK treatments, respectively. Under drought stress, the soluble sugar (SS) content in wheat increased, with the SS content under sorbitol-chelated potassium treatment being 8.30%, 16.38%, and 16.69% higher than those under S, K, and MK treatments, respectively.

### 3.2 Metabolomic profiles of wheat under drought stress

#### 3.2.1 Principal component analysis

**Figure 6:**
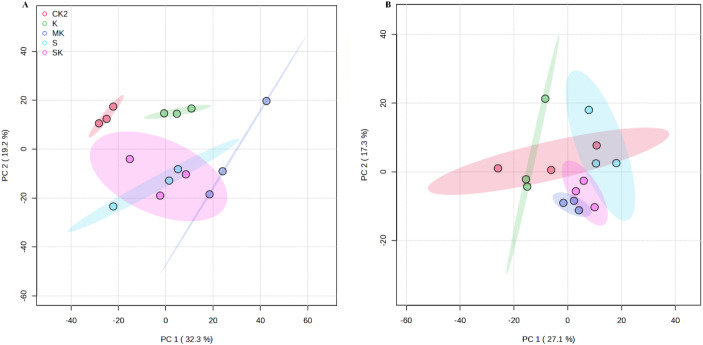
The PCA results of metabolites in wheat leaves. **(A)** Positive ion mode. **(B)** Negative ion mode.

PCA was performed to compare metabolic differences among the treatment groups (SK, K, MK, S, and CK_2_) and assess the overall metabolomic changes in wheat seedling leaves under drought stress. Additionally, the repeatability and stability of metabolites within each treatment group were evaluated. **Figures 6A** presents the PCA results for positive and negative ion modes. The total explained variances were 51.5% and 44.4%, respectively, indicating satisfactory clustering of samples. In both modes, the metabolite profiles of potassium chloride (K) and sorbitol-chelated potassium (SK) treatments were clearly separated, suggesting distinct metabolic responses. While partial overlap was observed between sorbitol-chelated potassium (SK) and potassium mixed with sorbitol (MK) treatments, overall significant differences persisted, indicating that the three potassium formulations exerted differential effects on the metabolism of wheat seedling leaves. These metabolic distinctions were consistent with the variations in agronomic traits, physiological parameters, and biochemical indices described as described above.

#### 3.2.2 Identification of differentially expressed metabolites

**Figure 7:**
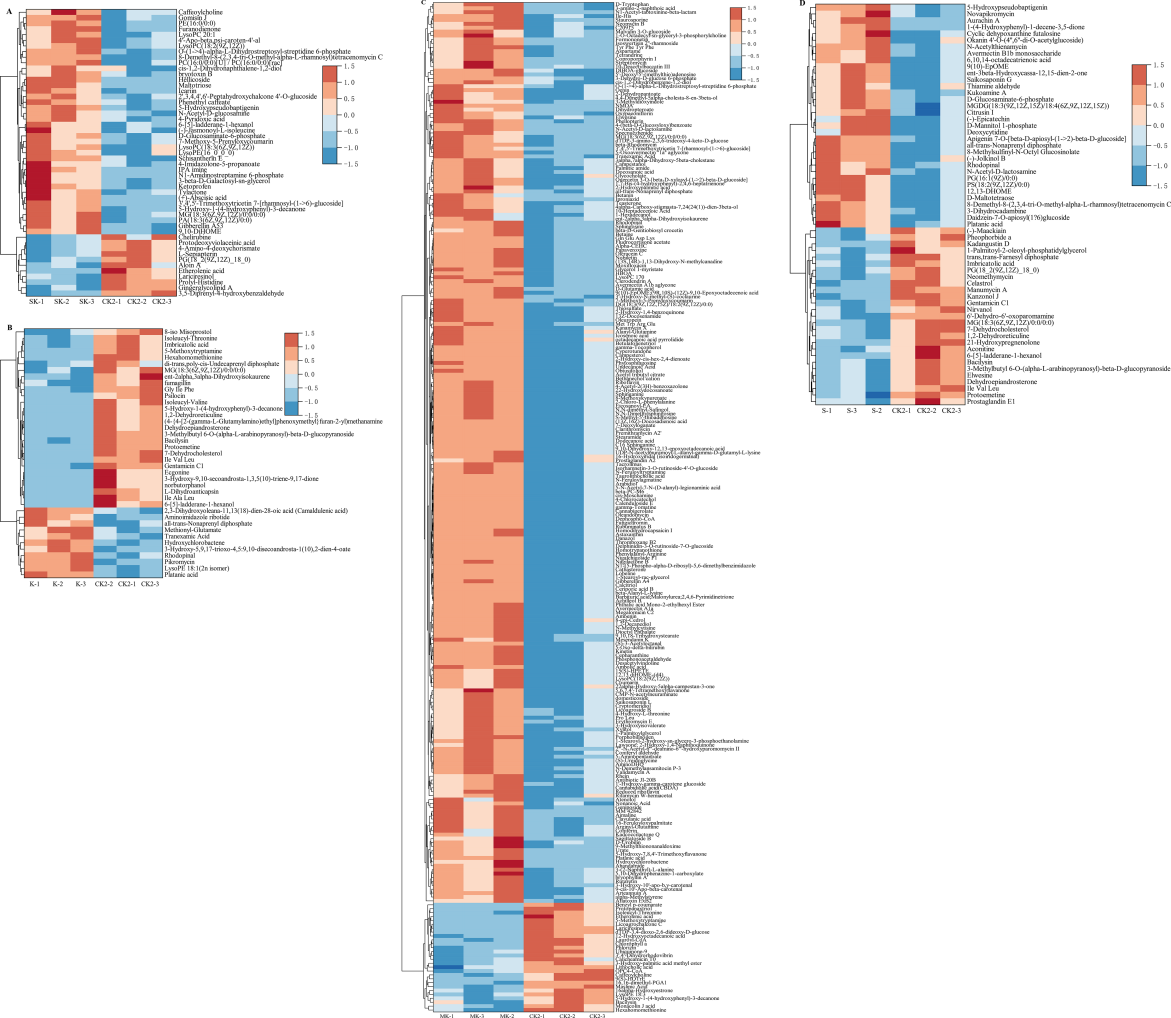
Heat map analysis of metabolites identified under drought stress compared with CK_2_. **(A)**SK.VS.CK_2_; **(B)** K.VS.CK_2_; **(C)** MK.VS.CK_2_; **(D)** S.VS.CK_2_. .

Using the screening criteria of P-value < 0.05, projection (VIP) value of > 1, and a fold change≥2 or ≤0.5, a total of 418 differential metabolites (DEMs) were identified among 1,788 metabolites with known structures (1,243 identified in the positive ion mode and 545 in the negative ion mode) (**Supplementary Material 1**). All experiments were performed with three biological replicates to ensure data reliability. Among them, 51 DEMs (11 upregulated and 40 downregulated) were identified in SK vs. CK_2_; 62 DEMs (30 upregulated and 32 downregulated) in K vs. CK_2_; 259 DEMs (28 upregulated and 231 downregulated) in MK vs. CK_2_; and 42 DEMs (8 upregulated and 34 downregulated) in S vs. CK_2_ (**Figure 7**). The results indicated that, compared with CK_2_, the number of downregulated DEMs affected by drought stress was greater than that of upregulated ones across all treatments. To clarify the characteristics of DEM expression in sorbitol-chelated potassium, upregulated metabolites (FC≥2) in SK.VS.CK_2_ were found to include Prolyl-Histidine, Lariciresinol, Chelirubine, L-Sepiapterin, 3,5-Diprenyl-4-hydroxybenzaldehyde, Etherolenic acid and 4-Amino-4-deoxychorismate, while downregulated metabolites (FC ≤ 0.5) included (-)-Jasmonoyl-L-isoleucine, N-Acetyl-D-glucosamine, (+)-Abscisic acid, 5-Hydroxypseudobaptigenin, and D-Glucosaminate-6-phosphate (**Supplementary Material 2**).

#### 3.2.3 Functional annotation and enrichment analysis of differential metabolites based on KEGG

The DEMs from each comparison group were matched to the KEGG database to obtain information on the metabolic pathways in which these metabolites are involved. Based on the adjusted P-value (P adjust) < 0.05, KEGG enrichment analysis was performed on the annotated results to further identify differences in metabolite expression among treatments. The results showed that, in the comparisons of SK.vs. CK_2_, MK.vs. CK_2_, K.vs. CK_2_, and S.vs. CK_2_, the DEMs were annotated to 18, 24, 16, and 61 metabolic pathways, respectively (**Supplementary Material 3**).

**Figure 8:**
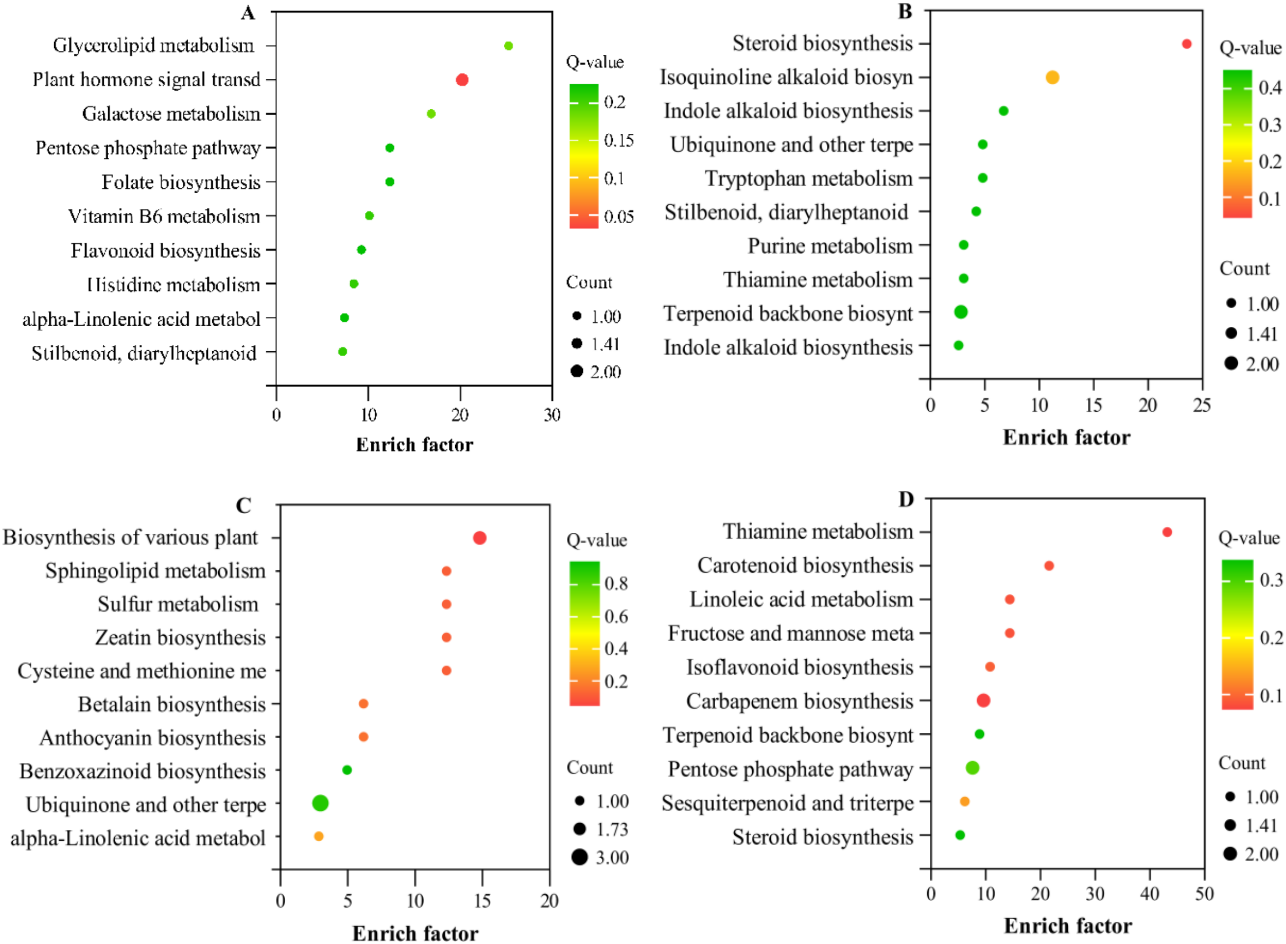
KEGG analysis of major metabolic pathways under drought stress compared to CK_2_. The size and shade of the circles represent the number of differentially expressed metabolites and P-values (P adjust), respectively. **(A)** SK.VS.CK2; **(B)** K.VS.CK2; **(C)** MK.VS.CK2; **(D)** S.VS.CK_2_.

Selection of 10 metabolic pathways significantly enriched in DEMs (**Figure 8**). Compared to CK_2_, foliar spraying with SK, K, MK, and S activated a broad range of metabolites, particularly those involved “antioxidation” and regulating “osmotic potential” (Mukarram et al., 2021), so their foliar application mitigated drought-induced damage in wheat seedlings (**Figures 3**, **4**). In contrast, the SK, K, MK, and S mobilized the most diverse set of metabolites. Specifically, in SK.VS.CK_2_, upregulated metabolites were primarily enriched in metabolic pathways such as Folate biosynthesis、alpha-Linolenic acid metabolism, while downregulated metabolites were mainly enriched in pathways such as Glycerolipid metabolism、Plant hormone signal transduction and Galactose metabolism Metabolic pathway enrichment analysis revealed that SK application modulated key metabolic pathways associated with intracellular water balance, ROS scavenging, and overall seedling vigor, thereby improving drought tolerance in wheat.

### 3.3 The correlation of wheat growth with physiological and biochemical indices, and differential metabolites

**Figure 9:**
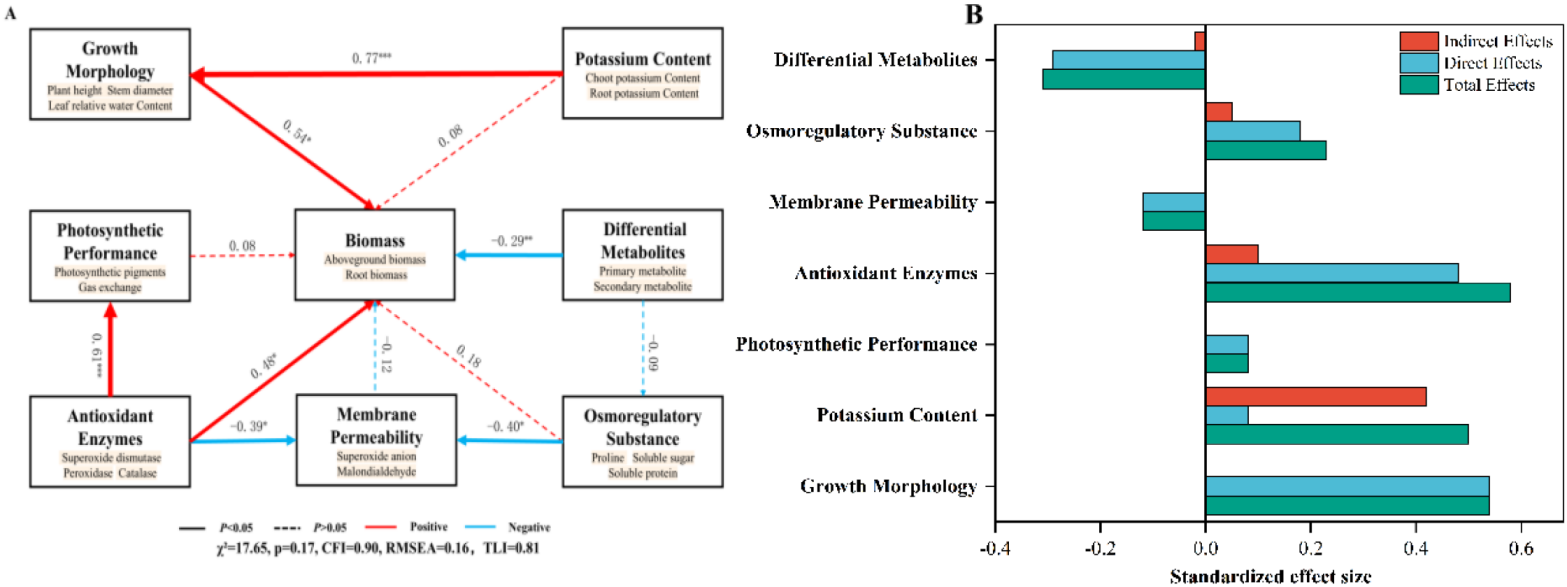
**(A)** The relationship between wheat biomass and physiological and biochemical parameters. **(B)** Summed direct effect and indirect effects. Note: *, **, *** indicate significant difference at the level of 0.05, 0.01, 0.001, respectively.

To further elucidate the underlying mechanisms of these effects, a structural equation model (SEM) (**Figure 9**) was employed to quantify the direct and indirect effects of six factors—growth morphology, potassium content, photosynthetic performance, antioxidant enzymes, membrane permeability, osmotic regulatory substances, and differential metabolites—on biomass accumulation. The SEM results indicated a good model fit, with a model χ² value of 17.65, a P value of 0.17, a CFI of 0.90, an RMSEA of 0.16, and a TLI of 0.81, all of which met the standard criteria. The SEM analysis demonstrated that all six factors exerted either direct or indirect effects on biomass accumulation. The direct effect values of growth morphology, potassium content, photosynthetic performance, antioxidant enzymes, membrane permeability, osmotic regulatory substances, and differential metabolites on biomass were 0.54, 0.08, 0.08, 0.48, -0.12, 0.18, and -0.29, respectively, among which photosynthetic performance, antioxidant enzymes, and differential metabolites showed the most pronounced effects. Both antioxidant enzyme activity and osmotic regulatory substances exhibited significant direct negative effects on membrane permeability, with comparable magnitudes (effect values of −0.39 and −0.40, respectively). In addition, there is a portion of the influence that is indirectly generated through mediating variables. Potassium content exerted an indirect positive effect (0.42) on biomass accumulation through the mediating variable of growth morphology, resulting in a total effect value of 0.50.

## 4 Discussion

### 4.1 Exogenous sorbitol-chelated potassium affects growth and photosynthetic performance of wheat seedlings under drought stress

Drought stress inhibits wheat seedling growth and biomass accumulation, reduces photosynthetic pigment content, and directly impairs net photosynthetic efficiency and stomatal conductance. The results of this study indicate that wheat plants treated with sorbitol-chelated potassium exhibited significantly higher leaf potassium content compared to those treated with potassium chloride, accompanied by improved photosynthetic performance. This effect may be attributed to sorbitol serving as a chelating ligand, which enhances potassium accumulation and utilization efficiency within the plants, thereby effectively alleviating growth inhibition of wheat seedlings under drought stress. Drought stress impedes the synthesis of photosynthetic pigments in wheat seedling leaves, thereby reducing photosynthetic capacity. Chlorophyll, a key pigment for light energy absorption, plays a key role in regulating photosynthetic efficiency (Bano et al., 2021). In this study, SK treatment significantly increased total chlorophyll content compared with non-chelated MK treatment, and this change was positively correlated with the net photosynthetic rate. These findings suggest that SK helps inhibit chlorophyll degradation, thereby maintaining photosynthetic stability under drought stress.

To cope with water deficit, plants typically adjust their stomatal aperture to reduce transpiration, limiting gas exchange and preventing excess water loss (Mukarram et al., 2021). In this study, SK treatment reduced stomatal conductance, indicating enhanced drought resistance through partial stomatal closure. This effect may reflect sorbitol-chelated potassium induced regulation of stomatal behavior near its physiological threshold. Drought stress leads to a decrease in Pn, which is attributed on the one hand to stomatal limitations and on the other hand to non-stomatal limitations (NSLs) (Dong et al., 2025). Stomatal limitation refers to the obstruction of CO_2_ diffusion through stomata into the leaf interior; NSL refers to the obstruction of CO_2_ diffusion within mesophyll cells (Grassi and Magnani, 2005). Under drought stress, Gs decreases while Ci increases, which may be dominated by NSL (**Figure 3**) The results of this study confirm the early conclusion that drought causes NSL (Salmon et al., 2020). Stomata are restricted in reducing transpiration while also limiting the entry of CO_2_. Research indicates that a decline in intercellular carbon dioxide concentration reduces photosynthetic activity, consequently impairing plant growth and development (Abobatta, 2020). By converting free potassium ions into organically chelated forms, sorbitol-chelated potassium enhances photosynthetic efficiency and facilitates the transport of photosynthetic products, thereby promoting crop growth and development. Therefore, chelated potassium may enter the leaf interior more easily by penetrating the lipid bilayer of the leaf surface cuticle and cutin due to the lipophilicity of its chelate, and exhibit superior mobility in the phloem. In this study, sorbitol-chelated potassium not only regulated wheat seedling growth but also significantly improved photosynthetic performance and activated antioxidant enzyme activity. These findings provide an important foundation for a deeper understanding of the comprehensive physiological mechanisms by which sorbitol-chelated potassium alleviates drought stress.

### 4.2 Effects of exogenous sorbitol-chelated potassium on antioxidant activity and osmoregulation in wheat seedlings under drought stress

Drought stress leads to an increase in superoxide anion and malondialdehyde content in wheat seedlings, disrupting the dynamic balance of reactive oxygen species (ROS). MDA, a well-established marker of lipid peroxidation, was significantly elevated in CK_2_ treatment, consistent with the findings of Aksu and Altay (2020). Compared to MK treatment, the application of SK significantly reduced MDA levels, suggesting that SK enhances ROS scavenging capacity. This effect may be attributed to the chelating action of sorbitol, which alters the form of potassium ions, to facilitate its absorption by the leaf surface. This mechanism is comparable to how sorbitol facilitates calcium transport (Li T. S. et al., 2024), contributing to improved drought stress mitigation in wheat plants. Under drought stress, plants alleviate oxidative damage caused by reactive oxygen species by enhancing antioxidant enzyme activity on one hand (Choudhury et al., 2017), and maintain cellular homeostasis by accumulating osmolytes on the other.

Osmotic adjustment (OA), as a plant defense mechanism, can enhance plant drought tolerance (Shao et al., 2005). Compared with inorganic potassium treatment, wheat seedling leaves treated with sorbitol-chelated potassium exhibited stronger osmotic adjustment capacity, with significant increases in Pro, SS, and SP contents, which may be closely related to enhanced plant stress resistance. However, as the intensity or duration of the stress increases, the regulatory capacity of the plant may surpass its threshold, leading to dysfunction of the antioxidant defense system, intensified lipid peroxidation of the cell membrane, and ultimately, irreversible damage to the plant (Azarabadi et al., 2017). In this study, drought stress was found to decrease the activity of SOD, POD, and CAT, while increasing O_2_^-^ and MDA content, suggesting that the damage inflicted on wheat leaves by drought stress surpassed the protective capabilities of the antioxidant enzyme system. Conversely, foliar application of SK treatment (compared to CK_2_) significantly enhanced antioxidant enzyme activities, suggesting that sorbitol-chelated potassium effectively boosts antioxidant defenses, thereby mitigating drought-induced damage in wheat. Building on these findings, metabolomic analysis provides additional insights into the molecular mechanisms through which SK modulates redox balance and enhances drought tolerance at the metabolic regulation level.

### 4.3 Effects of exogenous sorbitol-chelated potassium on differential metabolite expression of wheat seedlings under drought stress

KEGG enrichment analysis revealed that both metabolic pathways and biosynthetic pathways of secondary metabolites were highly enriched with differential metabolites, suggesting a close association between these pathways and wheat growth under water-limited conditions. In this study, a comparison with the CK_2_ treatment identified a total of 167 up-regulated and 170 down-regulated metabolites in the metabolic and biosynthetic pathways of secondary metabolites in the SK, K, MK, and S treatments. Compared with CK_2_ treatment, the K exhibited a higher number of upregulated differential metabolites than downregulated ones. Conversely, SK, MK, and S treatments showed a greater number of downregulated than upregulated metabolites. This pattern suggests that exogenous application of sorbitol-containing treatments primarily mediates drought stress responses through metabolite downregulation, whereas potassium applied alone (K) may mitigate drought-induced damage by promoting metabolite upregulation.

The upregulation of differential metabolites likely reflects an active adaptive response of wheat seedlings to environmental stress, enabling the enhancement of protective biochemical processes. Conversely, the downregulation of metabolites may represent a strategy for reducing overall metabolism, suppressing growth, and facilitating adaptation to unfavorable conditions. To mitigate drought stress, wheat synthesizes significant quantities of primary and secondary metabolites, including soluble sugars, amino acids, lipids, flavonoids, and other antioxidant compounds, while also activating detoxification enzymes (Raza et al., 2025). These differential metabolites highlight the mechanisms through which exogenous sorbitol-chelated potassium application mobilizes metabolic pathways in wheat leaves to coordinate physiological and biochemical responses, thereby alleviating drought-induced damage in wheat.

#### 4.3.1 Primary metabolites

Sugars serve as not only as a primary energy source for plants but also as key signaling molecules that regulate various physiological processes during growth and stress adaptation (Zhang et al., 2022). Varshney et al. (2023) revealed that celery and many Rosaceae species exhibit drought stress resistance primarily due to the accumulation of sorbitol and mannitol, which function as osmotic protectants and antioxidants. Soluble sugars undergo rapid interconversion; sucrose is hydrolyzed into glucose and fructose, while these hexoses, in turn, promote sucrose resynthesis, thus integrating into multiple metabolic pathways (Saddhe et al., 2021). The results of this study indicate that during SK, MK, and S treatments the levels of D-glucosamine-6-phosphate and xylitol, which participate in glycolysis and gluconeogenesis via the pentose phosphate pathway, were downregulated. This downregulation may indicate an adaptive strategy to reduce energy consumption, as gluconeogenesis is energetically costly, thereby limiting energy loss under stress conditions. Previous studies suggest that sugar metabolism plays a dual role in regulating both energy balance and osmotic adjustment during drought. Furthermore, accumulating evidence indicates that amino acids also play a crucial role in plant stress resistance by functioning as osmotic regulators and signaling molecules, reinforcing their significance in drought tolerance mechanisms.

Amino acids function as osmolytes, precursors of secondary metabolites, ROS scavengers, and potential regulatory and signaling molecules that help plants to cope with stress. Elevated levels of specific amino acids are considered functionally significant in enhancing stress resistance (Hildebrandt, 2018). Drought stress typically induces a significant accumulation of free amino acids in wheat seedlings, which may contribute to improved osmotic stress tolerance. These findings align with previous reports in other plant species. In this study, we observed significant changes in the metabolic pathways of several amino acids in wheat leaves under drought stress. Notably, pathways related to tryptophan, glutamate, valine, leucine, and isoleucine were significantly downregulated, while the free amino acid pools of proline, tryptophan, and branched-chain amino acids (valine, leucine, and isoleucine) were elevated (Guo et al.,2018; You et al., 2019). In our study, we found that the metabolic pathways of several high-abundance amino acids and most low-abundance amino acids in wheat leaves were significantly downregulated during drought stress, including those of tryptophan, glutamate, valine, leucine, and isoleucine, suggesting that wheat seedlings mitigate drought stress by accumulating free amino acids. Levels of amino acids, most notably proline, tryptophan, and the branched chain amino acids leucine, isoleucine, and valine were increased under drought stress in all cultivars. These findings suggest that wheat seedlings mitigate drought stress through the accumulation of amino acids, serving both as osmoprotectants and regulators of metabolic homeostasis.

Lipids and lipid-like molecules in plant cells are critical not only for membrane structure and energy storage but also for diverse biological functions. They serve as signaling molecules and as precursors for the synthesis of defense-related phytohormones, such as jasmonic acid (Suh et al., 2022). In this study, linolenic acid and α-linolenic acid metabolic pathways were significantly affected by the SK, MK, and S treatments. Compared with potassium application alone, sorbitol-containing treatments promoted greater lipid accumulation in wheat leaves, suggesting enhanced membrane integrity and stress signaling capacity. In addition, purine and pyrimidine metabolism, key components of plant energy metabolism (Gong et al., 2010), were modulated by SK application, contributing to the maintenance of energy homeostasis under drought conditions. Collectively, these findings demonstrate that exogenous SK application significantly enhances wheat drought tolerance by regulating carbohydrates, amino acid, and lipid metabolism, thereby improving energy metabolism, osmotic regulation, and cell defense mechanisms. These findings reveal the complexity and synergy of plant metabolic networks and provide an important foundation for further exploration of the role of secondary metabolites in drought resistance mechanisms in wheat seedlings.

#### 4.3.2 Secondary metabolites

Plant hormones are central regulators enabling plants to cope with environmental stress, integrating external drought signals with internal physiological responses. In this study, the formation of the jasmonoyl-isoleucine conjugate in the SK treatment likely contributed to stress resistance. Jasmonoyl-isoleucine levels typically increase under dehydration stress (Urano et al., 2017) and interact with abscisic acid (ABA) accumulation to participate in the dehydration signal transduction pathway (de Olllas et al., 2015; Wang et al., 2021). Therefore, sorbitol-chelated potassium may alleviate drought stress by mobilizing jasmonoyl-isoleucine and modulating hormone-mediated signaling in wheat leaves. Beyond hormonal signaling, carotenoids play a crucial role in drought tolerance as photoprotective agents, antioxidants, and precursors for ABA biosynthesis.

Carotenoid accumulation not only provides precursors for the biosynthesis of the plant hormone ABA (Yu et al., 2022; Imtiaz et al., 2023), but also enhances drought tolerance by supporting ROS scavenging and improving photoprotection (Demmig-Adams and Adams, 2002). In this study, Rhodopinal, Astaxanthin, and Hydroxychlorobactene were detected in the MK, S, and K treatments. As a carotenoid derivative, Rhodopinal and Hydroxychlorobactene possess characteristic chromophores that assist photosynthetic pigments and scavenge free radicals (Berry et al., 2019). Additionally, astaxanthin, a highly efficient antioxidant, was identified and may contribute to enhanced antioxidant capacity and stress adaptation (Wang K. et al., 2024). These findings suggest that carotenoids, as key components of the non-enzymatic antioxidant system, mitigate drought stress by scavenging ROS modulating detoxification enzyme activity. Similarly, flavonoids, another class of secondary metabolites, play multifaceted roles in plant responses to drought stress.

Flavonoids regulate hormone signal transduction and act as inhibitors of auxin transport (Peer and Murphy, 2007). Recent studies have shown that flavonoids, such as flavones, flavanones, flavonols, isoflavones, and anthocyanins, play an essential role as ROS scavengers, contributing significantly to oxidative stress mitigation (Brunetti et al., 2018). Furthermore, core members of flavonoids such as anthocyanins possess strong antioxidant and antimutagenic properties (Yang et al., 2021). In this study, anthocyanin biosynthesis was observed in MK-treated plants, suggesting a potential role for these compounds in drought tolerance mechanisms. Notably, the expression of some flavonoids was downregulated under drought stress, indicating a dynamic regulatory adjustment. This pattern suggests that the production of flavonoid-related metabolites contributes to plant defense against drought stress and positively influences growth and performance. Plant hormones, carotenoids, and flavonoids, key regulators in plant metabolic networks, work in coordination to mediate drought stress responses. Plant hormones regulate physiological processes primarily through signal transduction pathways, while carotenoids and flavonoids, as secondary metabolites, enhance stress resistance through photoprotection, antioxidation, and regulation of hormone signaling. Collectively, these integrated processes highlight the synergistic regulatory role of primary and secondary metabolites in improving plant drought stress tolerance, orchestrating these metabolic and signaling adjustments to optimize plant responses to water deficit.

### 4.4 Mechanism of analysis of exogenous sorbitol-chelated potassium treatment on biomass of wheat seedlings under drought stress

**Figure 10:**
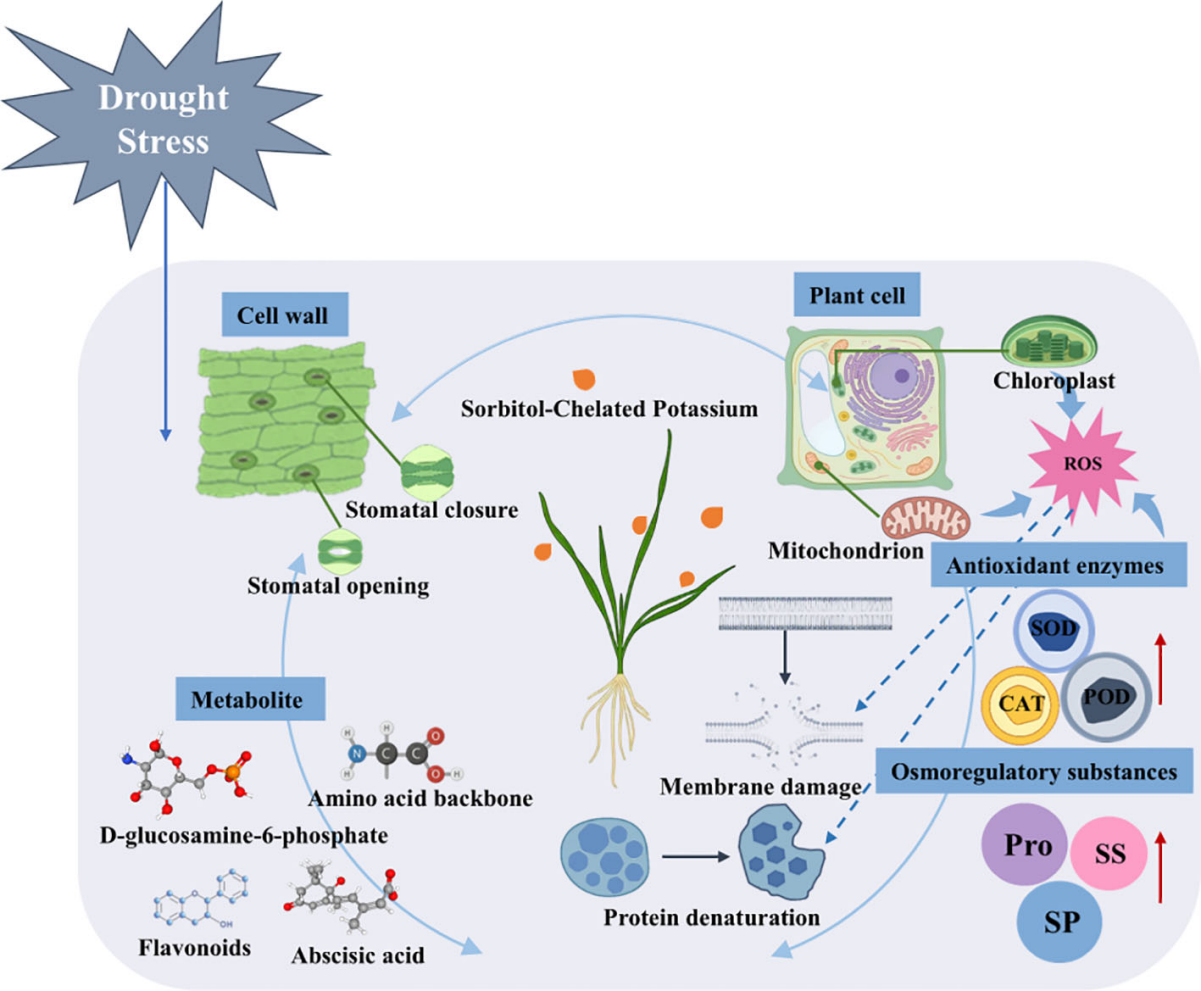
Schematic diagram of sorbitol-mediated potassium chelation to cope with drought stress. Created with BioRender (https://www.biorender.com/sub-categories/plant-anatomy), licensed under CC BY 4.0.

The drought resistance of wheat is jointly influenced by plant potassium content, photosynthetic performance, antioxidant system, osmotic regulation system, and metabolite expression; however, existing studies lack a comprehensive analysis integrating these factors. This study reveals the mechanism by which sorbitol-chelated potassium enhances plant stress resistance by regulating key endogenous physiological processes (**Figure 10**). Through structural equation modeling (SEM) analysis, its contribution was quantified: both growth morphology and enzyme activity showed significant positive correlations with biomass accumulation, with enzyme activity exhibiting the highest total effect value (0.58). This finding is consistent with the results presented in Section 3.2.3, which demonstrated that sorbitol-chelated potassium regulates differential metabolites involved in antioxidant processes. These results indicate that exogenous application of sorbitol-chelated potassium can enhance the antioxidant capacity of wheat, thereby improving its drought resistance. Under drought stress, this treatment simultaneously activates relevant enzyme activities and promotes the synthesis of osmotic regulatory substances while synergistically reducing the accumulation of ROS. Metabolomic analysis revealed that the number of downregulated metabolites was substantially greater than that of upregulated ones. In combination with the SEM results (differential metabolites–biomass accumulation), this study further demonstrated that wheat may downregulate certain metabolites to reduce excessive consumption of assimilates and energy, thereby adjusting metabolic status and enhancing drought adaptation (Dhanyalakshmi, et al., 2025). Consequently, exogenous application of sorbitol-chelated potassium alleviated the inhibitory effects of drought stress by strengthening endogenous factors and coordinately regulating differential metabolites such as D-glucosamine-6-phosphate and (-)-jasmonoyl-L-isoleucine. This mechanism provides new insights into the integrated study of growth and metabolic regulation in wheat seedlings under adverse conditions.

In summary, this study used PEG-6000 to simulate drought conditions. Although this method allows for rapid and controllable simulation of water stress, differences still exist compared to field soil drought in terms of water gradients, root-microbe interactions, and chemical signal transmission. Furthermore, whether chelated potassium has stronger mobility than free potassium needs to be directly verified through experiments on cuticular permeability rate separation and phloem transport monitoring. In the future, we will further validate the key metabolites regulating leaf growth in plants through soil drought experiments. By delving into the physiological mechanisms of plants under different water gradients, we aim to elucidate the mechanisms by which exogenous spraying of SK enhances crop drought tolerance from the perspectives of transcriptomics, root exudates, and plant nutrition. This research can provide a theoretical basis for developing agronomic measures to alleviate crop stress and lay the foundation for advancing research in the field of drought resistance.

## 5 Conclusion

The exogenous application of sorbitol-chelated potassium (SK) alleviates drought-induced growth inhibition in wheat seedlings through a multi-faceted physiological mechanism. This mitigation is achieved by enhancing photosynthetic pigment content and gas exchange capacity, upregulating antioxidant enzyme activity, and regulating accumulation of key metabolites. Drought stress significantly limits wheat growth and biomass accumulation. Compared to CK_2_, SK application significantly mitigated biomass reduction (19.2%) under drought stress conditions. Exogenous SK significantly increased photosynthetic pigment content and gas exchange parameters in wheat flag leaves, while enhanced antioxidant enzyme activity, reduced reactive oxygen species levels, and promoted the synthesis of osmolytes, significantly alleviating oxidative damage caused by drought stress. Untargeted metabolomics analysis revealed that SK treatment induced the relative accumulation of differential metabolites, thereby inhibiting sugar metabolism to minimize energy loss, accumulating free amino acids to enhance osmotic adjustment, and promoting lipid metabolism to maintain cellular energy homeostasis. Additionally, SK mitigated drought-induced growth inhibition in wheat by modulating plant hormone signaling, enhancing carotenoid and flavonoid biosynthesis, and activating detoxification enzyme system to scavenge ROS.

## Data Availability

The original contributions presented in the study are included in the article/**supplementary material**. Further inquiries can be directed to the corresponding author.

## Supplementary material

Supplementary Material 1 Metabolites were identified through comparative analysis of various spraying treatments against the control group (CK_2_).

Supplementary Material 2 Differentially expressed metabolites were screened by comparing various spraying treatments with the control group (CK_2_).

Supplementary Material 3 Functional annotation and enrichment analysis of differential metabolites based on KEGG.
